# Biological Resectability in Colorectal Liver Metastases: A Nomogram‐Based Continuous Risk Model to Define Borderline Disease

**DOI:** 10.1002/ags3.70278

**Published:** 2026-09-08

**Authors:** Yuzo Umeda, Toshiharu Mitsuhashi, Tomokazu Fuji, Toru Kojima, Takeshi Koujima, Tatsuo Matsuda, Daisuke Satoh, Masaru Inagaki, Kosei Takagi, Toshiyoshi Fujiwara

**Affiliations:** ^1^ Department of Hepatobiliary Pancreatic and Transplantation Surgery Ehime University Graduate School of Medicine Matsuyama Ehime Japan; ^2^ Department of Gastroenterological Surgery Okayama University Graduate School of Medicine, Dentistry and Pharmaceutical Sciences Okayama Japan; ^3^ Center for Innovative Clinical Medicine, Medical Development Field Okayama University Okayama Japan; ^4^ Department of Surgery Okayama Saiseikai General Hospital Okayama Japan; ^5^ Department of Surgery Himeji Red Cross Hospital Himeji Japan; ^6^ Department of Surgery Tenwakai Matsuda Hospital Kurashiki Japan; ^7^ Department of Surgery Hiroshima Citizens Hospital Hiroshima Japan; ^8^ Department of Surgery National Hospital Organization Fukuyama Medical Center Fukuyama Japan

**Keywords:** biology, borderline resectable, colorectal liver metastases, genetic profile, KRAS, morphology

## Abstract

**Background:**

Resectability in colorectal liver metastases (CRLM) is traditionally defined by technical feasibility; however, a biologically based definition of borderline resectable disease has not been established. We evaluated the prognostic impact of *KRAS* mutation and developed a model for biological risk stratification.

**Methods:**

This multi‐institutional retrospective study included 584 patients undergoing initial hepatectomy for CRLM. The impact of *KRAS* mutation was assessed according to primary tumor sidedness and tumor burden score (TBS). A nomogram predicting overall survival (OS) was constructed using independent prognostic factors and compared with established clinical risk scores.

**Results:**

*KRAS* mutation was identified in 36.8% of patients and was associated with worse OS (median survival: 47.3 vs. 70.6 months, *p* = 0.0006) and recurrence‐free survival. This adverse impact was observed in left‐sided tumors but not in right‐sided tumors. TBS stratified prognosis in *KRAS* wild‐type but not in mutant cases, suggesting that tumor biology may outweigh morphology. A nomogram incorporating *KRAS* status, TBS, CA19‐9, primary nodal status, and extrahepatic disease demonstrated improved discriminatory ability compared with conventional models and stratified recurrence patterns, including extrahepatic recurrence.

**Conclusions:**

The prognostic impact of *KRAS* mutation depends on tumor sidedness and is independent of tumor morphology. Our nomogram provides a continuous biological risk assessment and may facilitate biological risk stratification and support future refinement of the concept of biologically borderline resectable CRLM.

## Graphical abstract

*KRAS* mutation demonstrated a prognostic impact according to primary tumor sidedness, independent of tumor morphology, in patients undergoing hepatectomy for colorectal liver metastases. A nomogram integrating biological and clinicopathological factors was developed to provide continuous biological risk assessment. The proposed model may facilitate individualized prognostic stratification and support future refinement of the concept of biological resectability.

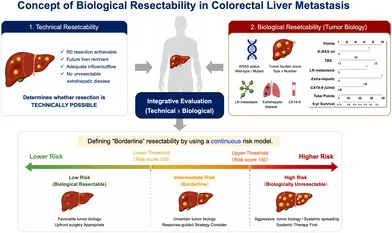

**Abbreviations**
AIC: Akaike's Information Criterion
BR: borderline resectable
CA19‐9: carbohydrate antigen 19‐9
CEA: carcinoembryonic antigen
CRC: Colorectal cancer
CRLM: Colorectal liver metastasis
HR: hazard ratio
IQR: interquartile range
*KRAS*
: Kirsten rat sarcoma
MST: median survival time
OS: overall survival
RFS: recurrence‐free survival
TBS: Tumor burden score
TP: total points

## 1 Introduction

Colorectal cancer (CRC) is a leading oncological cause of morbidity and mortality worldwide. Surgery is the treatment of choice for patients with localized disease; however, over half of all patients develop metastases and the liver is the most frequent site of CRC metastasis [1]. Patients with colorectal liver metastases (CRLM) have poor prognosis; with a median survival time in untreated patients of only 8 months [2]. Various approaches have been used in the treatment of CRLM, including surgery, chemotherapy, radiofrequency ablation, radiotherapy, as well as the combination of these modalities. Surgical resection is currently considered the first‐line treatment that offers a chance of cure and long‐term survival [3]. However, there are cases of early recurrence and poor prognosis even after hepatic resection.

The selection criteria for hepatic resection of CRLM include controlled primary tumor, no extrahepatic metastases, and technically feasible resection with tumor‐free margins [4]. The combination of chemotherapy, which has made remarkable progress in recent years, and surgical resection is expected to expand the surgical indications and improve the prognosis of CRLM. To further improve the prognosis after such treatment, it is desirable to establish a risk model that helps in the selection of the most appropriate treatment modalities, especially liver resection.

In the era of targeted molecular therapy, molecular profiling of the Kirsten rat sarcoma (*KRAS*) oncogene has become indispensable for designing treatment strategies for CRLM [5]. In this regard, CRLM with *KRAS* mutations is reported to be associated with poor overall (OS) and recurrence‐free survival (RFS) after liver resection [5, 6]. In contrast, the correlation between *KRAS* mutations and long‐term prognosis is controversial; with some reports failing to confirm the prognostic value of *KRAS*. In this context, new and promising prognostic models that include *KRAS* status and traditional risk models have been developed [7, 8, 9]. Using such models, it seems that the anatomical location of the primary CRC is an important prognostic factor. In fact, several studies have demonstrated that the incidence, etiology, genetic background, and oncologic outcome are different in patients with right‐ and left‐sided CRCs [10].

The aim of this retrospective study was to determine the prognostic value of *KRAS* mutation, especially with regard to tumor sidedness and tumor morphology. We also developed a prognostic nomogram integrating molecular, morphological, and clinicopathological factors and explored whether this framework could provide continuous biological risk assessment relevant to the concept of biological resectability in CRLM.

## 2 Methods

### 2.1 Study Subjects

This multicenter retrospective study included 1251 adult patients with CRLM who underwent initial liver resection with curative intent between January 2006 and December 2016. The clinical data of these subjects were collected from 18 medical institutions across Japan (Okayama University Hospital, Okayama Saiseikai General Hospital, Hiroshima City Hiroshima Citizens Hospital, Kochi Health Sciences Center, Himeji Red Cross Hospital, National Hospital Organization Fukuyama Medical Center, Tottori Municipal Hospital, Tenwakai Matsuda Hospital, National Hospital Organization Okayama Medical Center, Fukuyama City Hospital, Himeji St. Maria Hospital, Matsuyama Shimin Hospital, National Hospital Organization Iwakuni Medical Center, Tsuyama Chuou Hospital, Okayama Rosai Hospital, Kagawa Prefectural Chuo Hospital, Kagawa Rosai Hospital, and Japanese Red Cross Kobe Hospital). Of these, 13 were board‐certified training institutions as qualified by the Japanese Society of Hepato‐Biliary‐Pancreatic Surgery. Consequently, most patients were recruited from high‐volume centers, which resulted in assured operative procedures and outcomes. The following exclusion criteria were applied: (1) unknown *KRAS* mutation profile (*n* = 649), (2) R2 resection as palliative liver resection (*n* = 14), (3) insufficient follow‐up data (*n* = 2), and (4) *BRAF* mutation (*n* = 2).

Thus, 584 participants were included in the study. The male: female ratio was 1.4:1. The median age was 66 years (interquartile range [IQR] 59–72), and 186 (31.8%) patients were over 70 years of age. With regard to the anatomic location of the primary CRC, 147 (25.2%) patients had right‐sided tumors while 437 (74.8%) had left‐sided tumors. Metastases to the regional lymph nodes were identified in 383 (65.6%) patients. Clinically, 396 patients were considered to have synchronous CRLM (disease‐free interval of less than 12 months) while the remaining 188 patients had metachronous CRLM. At the time of diagnosis of CRLM, extrahepatic metastatic disease was identified in 106 (18.1%) patients. The median number and maximum tumor size of CRLM at diagnosis were 2 (IQR 1–4) and 2.6 cm (IQR 1.7–4.2), respectively. CRLM were classified as solitary (*n* = 236, 40.4%), 2–4 (*n* = 211, 36.1%), and ≥ 5 (*n* = 137, 23.5%). In the pathological examination of the excised specimens, a margin width of ≥ 1 mm was defined as R0 resection, while a margin width of less than 1 mm was defined as R1 resection. After surgery, the median follow‐up period was 44.1 months (IQR 27.1–64.9). At the time of this study, the observation period was > 5 years in 82 (31.1%) patients, and > 10 years in 15 (2.6%) patients. During the follow‐up period, 462 (79.1%) patients developed tumor recurrence.

### 2.2 Assessment of Tumor Burden Score and KRAS Mutation

The tumor burden score (TBS) is a new marker of the cumulative impact of tumor size and number on survival [11]. It is calculated by assigning coordinates on a Cartesian plane to each patient according to the maximum tumor size (abscissa) and number of tumors (ordinate) at diagnosis of CRLM. The Pythagorean theorem is then used to calculate the distance (absolute value) of any point (corresponding to a patient with a particular tumor size and number) from the origin (0, 0) of the plane, so that TBS^2^ = [maximum tumor size]^2^ + [number of liver lesions]^2^. The estimated TBS values are classified by grades and correlate with progressively worse overall survival (OS). The median TBS score was 4 (IQR 3–7). The TBS was categorized into three grades: low: TBS < 3 (*n* = 169, 28.9%), intermediate: TBS 3–8 (*n* = 329, 56.3%), and high: TBS ≥ 9 (*n* = 86, 14.7%) [11].

All tumor tissue samples were formalin‐fixed and paraffin embedded after surgical resection. Genomic DNA was isolated from primary CRC or CRLM tissue specimens. Subsequently, *KRAS* mutations in codons 12 and 13 were analyzed using Sanger sequencing, as described in detail previously [12]. *KRAS* mutation analysis was performed using primary colorectal tumor specimens in 69% of patients and liver metastasis specimens in 31%, according to tissue availability and institutional practice. In the latter part of the study period, *NRAS* mutations in codons 12, 13, and 61 were also analyzed in addition to *KRAS* mutations. For the purpose of statistical analysis, *NRAS* mutant tumors were classified together with *KRAS* mutant tumors.

### 2.3 Statistical Analysis and Ethical Considerations

To evaluate the clinical impact of *KRAS* mutation on CRLM, differences between clinical variables were tested by the Mann–Whitney *U* test for continuous variables and Spearman's coefficients for categorical data. Continuous variables are presented as medians and IQR. Moreover, the cut‐off values for continuous variables were determined using ROC or obtained from previous studies. Statistical significance was set at *p* < 0.05. Survival curves were estimated using the Kaplan–Meier method for survival analysis, and differences in survival were evaluated using the log‐rank test. In addition, we identified the risk factors for oncological outcomes using the Cox proportional hazards model. Clinical variables with *p* values of < 0.05 in univariate analysis were entered into the multivariate analysis. A nomogram was developed to graphically illustrate the Cox proportional hazard regression model. In addition, a nomogram was developed using independent risk factors in the preoperative set to predict OS at the time of CRLM diagnosis to design a treatment strategy. To facilitate clinical interpretation, patients were categorized into three risk groups according to total nomogram points. These thresholds were selected based on the distribution of total points and the observed separation of survival curves and were intended for descriptive risk stratification rather than formal biological classification. Moreover, the discriminatory abilities of our new model and traditional prognostic models, including the Fong score and MD Anderson modified CRS clinical risk score (mCRS), were evaluated using Harrell's C‐index and Akaike's information criterion (AIC), and Bayesian information criterion (BIC). Differences in Harrell's C‐indices between the proposed model and each reference model were tested using two‐sided Wald‐type tests based on linear contrasts, with jackknife standard errors accounting for the correlation between C‐indices estimated within the same cohort. Finally, recurrence patterns after the initial hepatectomy in each risk model were compared using Spearman's coefficients. Internal validation of the Cox proportional hazards model used to construct the nomogram was performed using 1000 bootstrap resamples. Model discrimination was evaluated using Harrell's C‐index, and optimism‐corrected performance was estimated by bootstrap validation. Model calibration was assessed using bootstrap calibration plots for 3‐ and 5‐year overall survival. All statistical analyses were performed using Stata 17/MP (StataCorp LP, College Station, TX) and the R software (version 3.5.3).

The study was approved by the Ethics Committee of Okayama University Hospital. The need for a signed consent form was waived based on the retrospective nature of the study.

## 3 Results

*KRAS* mutations were detected in 215 (36.8%) patients, and the remaining 369 (63.2%) patients were identified as the wild type. Table S1 summarizes the differences between the *KRAS* mutation and wild‐type groups. The median age and proportion of elderly patients with *KRAS* mutations were higher than those of the wild‐type patients. In addition, *KRAS* mutations were more frequent in the right‐sided colon cancer patients than those with left‐sided colorectal cancer (*p* < 0.001). There were no significant differences in the other clinical variables (e.g., extrahepatic disease, primary nodal metastasis, tumor differentiation, tumor markers, and morphology of CRLM).

Survival analysis using data of the entire cohort showed that *KRAS* mutation correlated with shorter RFS compared to the wild type; the median survival time (MST) and 1−/3−/5‐year RFS rates were 9.6 months and 40.9/18.6/16.3% in *KRAS* mutation, 12.4 months and 51.7/25.7/21.9% in wild type, respectively (*p* = 0.044). Furthermore, *KRAS* mutation was associated with poorer OS than the wild type; the MST and 1−/3−/5‐year OS rates were 47.3 months and 93.5/65.0/36.8% in *KRAS* mutation, 70.6 months and 95.3/71.9/55.6% in the wild‐type, respectively (*p* = 0.0006, Figure S1).

**FIGURE 1:**
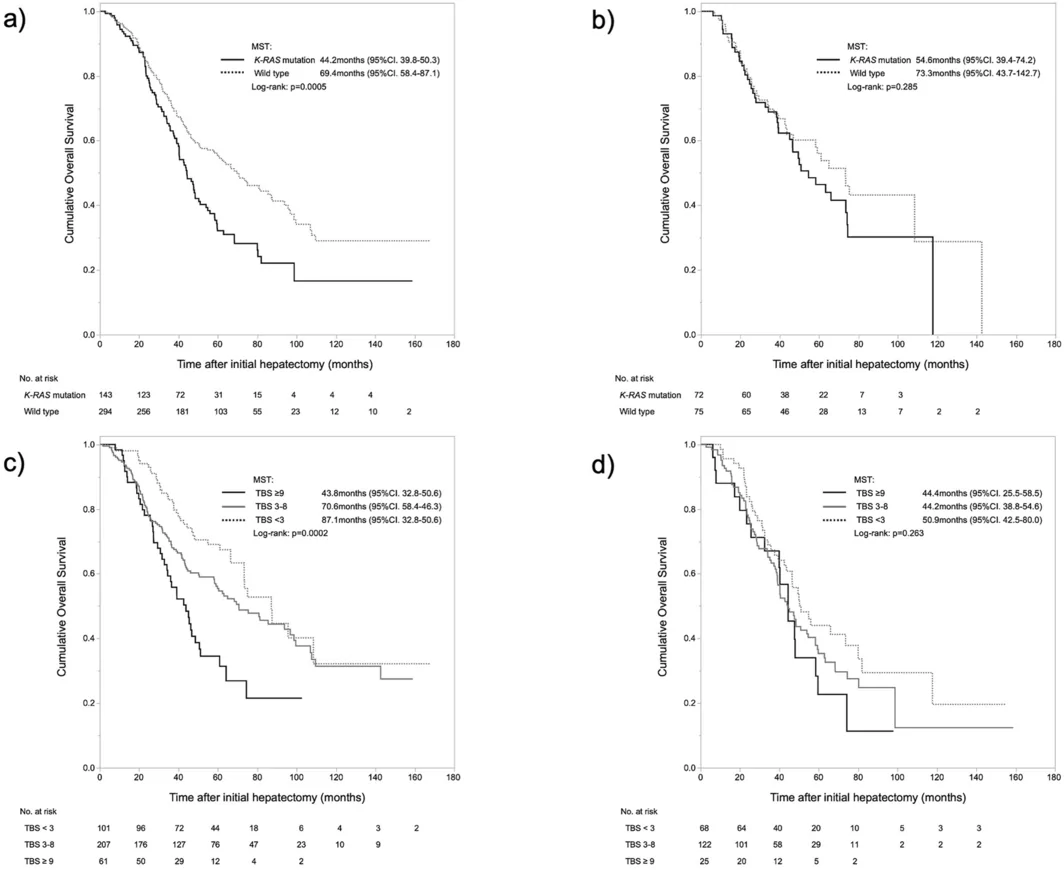
The Kaplan–Meier survival curves for overall survival (a) primary left sided CRC stratified by *KRAS* status (*n* = 437), (b) primary right sided CRC stratified by *KRAS* status (*n* = 147), (c) wild type stratified by tumor burden score (*n* = 369), and (d) KRAS mutation stratified by tumor burden score (*n* = 215).

With regard to the primary location of colon cancer, the prognostic impact of *KRAS* mutation varied between right‐ and left‐sided cancers. In patients with left‐sided primary CRC, *KRAS* mutation in CRLMs (*n* = 143) was associated with shorter OS than wild‐type CRLMs (*n* = 294): the MST and 1−/3−/5‐year OS rates were 44.2 months and 93.7/63.4, and 32.1% in *KRAS* mutation, 69.4 months and 95.5/72.3/55.5% in wild type, respectively (*p* = 0.0005, Figure 1a). On the other hand, the mutation did not affect the OS in patients with right‐sided colon cancer: the MST and 1−/3−/5‐year OS rates were 54.6 months and 93.0/68.9/46.4% in *KRAS* mutation (*n* = 72), 73.3 months and 95.9/69.7/56.0% in the wild type (*n* = 75), respectively (*p* = 0.285, Figure 1b). Similarly, *KRAS* mutation was an adverse prognostic factor for RFS on the left side but not on the right side (left side: *p* = 0.035; right side: 0.553). With regard to tumor morphology, the classified TBSs stratified OS more than the number of tumors or tumor size did (Table 1). These stratifications of prognosis by TBS grading were also observed in wild‐type: the MST was 87.1 months in TBS < 3, 70.6 months in TBS 3–8, and 43.8 month in TBS ≥ 9 (*p* = 0.0002, Figure 1c). In contrast, the prognosis was not well stratified by TBS grade in *KRAS* mutations: the MST was 50.9 months in TBS < 3, 44.2 months in TBS 3–8, and 44.4 month in TBS ≥ 9 (*p* = 0.263, Figure 1d). The above results indicate that the effect of *KRAS* mutation on prognosis is independent of tumor morphology.

**TABLE 1:** Results of univariate analysis of perioperative risk associated with overall survival after hepatectomy for CRLM (*n* = 584).

Risk factors	*n* (%)	Overall survival		Univariate analysis		
		5 years (%)	Median (month)	HR	95% CI	*p*
Gender						
Female	243 (41.6)	44.4	62.9	1.00 (reference)		
Male	341 (58.4)	51.8	49.9	0.85	0.68–1.07	0.195
Age						
≤ 70 years	398 (68.2)	51.2	62.3	1.00 (reference)		
> 70 years	186 (31.8)	43.3	48.1	1.26	0.99–1.59	0.054
Sidedness of primary colon cancer						
Right	147 (25.2)	51.0	61.0	1.00 (reference)		
Left	437 (74.8)	48.0	58.4	1.02	0.78–1.31	0.900
Primary tumor nodal metastases						
No	201 (34.4)	60.5	80.0	1.00 (reference)		
Yes	383 (65.6)	42.4	48.1	1.63	1.28–2.09	< 0.001
Primary tumor differentiation						
Well differentiated	118 (20.2)	50.5	62.3	1.00 (reference)		
Moderately differentiated	414 (70.9)	49.9	59.8	1.02	0.78–1.35	0.867
Poorly differentiated/mucinous	52 (8.9)	35.6	44.6	1.43	0.94–2.18	0.098
*K‐Ras* mutation status						
Wild type	369 (63.2)	55.6	70.6	1.00 (reference)		
Mutation	215 (36.8)	36.8	47.2	1.47	1.18–1.85	< 0.001
Disease‐free interval (months)						
≥ 12	188 (32.2)	53.1	68.4	1.00 (reference)		
< 12	396 (67.8)	46.8	57.9	1.16	0.92–1.48	0.195
Extra‐hepatic disease						
No	478 (81.8)	54.3	66.5	1.00 (reference)		
Yes	106 (18.2)	22.6	36.7	2.08	1.60–2.71	< 0.001
Tumor distribution						
Unilobar	372 (63.7)	49.3	59.7	1.00 (reference)		
Bilobar	212 (36.3)	47.8	58.5	1.11	0.89–1.40	0.352
Number of tumors (at diagnosis)						
1	236 (40.4)	53.1	66.5	1.00 (reference)		
2–4	211 (36.1)	53.4	68.4	1.02	0.78–1.32	0.961
≥ 5	137 (23.5)	33.5	43.7	1.72	1.31–2.27	< 0.001
Maximum tumor diameter (at diagnosis)						
≤ 5 cm	478 (81.8)	50.4	61.0	1.00 (reference)		
> 5 cm	106 (18.2)	41.3	48.0	1.27	0.96–1.67	0.092
Tumor burden score						
Low (< 3)	169 (28.9)	58.9	73.5	1.00 (reference)		
Intermediate (3–8)	329 (56.3)	48.3	59.1	1.31	1.00–1.69	0.047
High (≥ 9)	86 (14.7)	30.7	44.4	1.97	1.40–2.77	< 0.001
CEA level (at diagnosis)						
≤ 20 ng/ml	361 (61.8)	54.8	69.0	1.00 (reference)		
> 20 ng/ml	223 (38.2)	39.2	45.4	1.33	1.06–1.66	0.014
CA19‐9 level (at diagnosis)						
≤ 40 U/ml	387 (66.3)	56.1	73.3	1.00 (reference)		
> 40 U/ml	197 (33.7)	34.6	44.3	1.64	1.30–2.05	< 0.001
Preoperative chemotherapy (before hepatectomy)						
No	340 (58.2)	53.4	68.3	1.00 (reference)		
Yes	244 (41.8)	42.4	45.4	1.45	1.16–1.80	0.001
Extent of liver resection						
Hr1 or less	457 (78.3)	52.6	63.3	1.00 (reference)		
Hr2 or larger	127 (21.7)	34.5	43.9	1.53	1.19–1.97	0.002
Open/Laparoscopic procedure						
Open	515 (88.2)	48.5	59.1	1.00 (reference)		
Laparoscopic	69 (11.8)	51.0	75.0	0.90	0.62–1.30	0.586
Curability and pathological resection margin						
R0 resection	520 (89.0)	50.5	60.9	1.00 (reference)		
Combined use of RFA	26 (4.5)	43.0	43.8	1.64	1.04–2.58	0.033
R1 resection	38 (6.5)	27.6	34.0	1.88	1.25–2.84	0.003
Blood loss						
≤ 1000 mL	497 (85.1)	49.0	59.4	1.00 (reference)		
> 1000 ml	87 (14.9)	46.8	43.4	1.18	0.87–1.59	0.293
Blood transfusion						
No	488 (83.6)	50.8	61.0	1.00 (reference)		
Yes	96 (16.4)	39.0	47.7	1.32	1.01–1.75	0.048
Adjuvant chemotherapy (post hepatectomy)						
No	252 (43.2)	45.5	58.0	1.00 (reference)		
Yes	332 (56.8)	51.1	61.0	0.89	0.71–1.11	0.300

Abbreviations: CA19‐9, carbohydrate antigen 19–9; CEA, carcinoembryonic antigen; CI, confidence interval; HR, hazard ratio; Hr1, sectionectomy; Hr2, hemihepatectomy; RFA, radiofrequency ablation.

Univariate analysis of OS showed that primary tumor nodal metastasis, *KRAS* mutation, extrahepatic disease, TBS, carcinoembryonic antigen (CEA), and carbohydrate antigen 19–9 (CA19‐9) levels at CRLM diagnosis, preoperative chemotherapy, extent of liver resection, curability of liver resection, and blood transfusion were significant risk factors (*p* < 0.05, Table 1). The CA19‐9 cut‐off value that showed maximum sensitivity and specificity in predicting poor OS was 40 U/mL and was employed in subsequent univariate and multivariate analyses. Moreover, the cut‐off for CEA was set to 20 ng/mL, based on a previous report [8]. Multivariate analysis identified primary tumor nodal metastasis (hazard ratio [HR] 1.46, *p* = 0.003), *KRAS* mutation (HR 1.51, *p* < 0.001), extrahepatic disease (HR 1.8, *p* < 0.001), and TBS (TBS 3–8. HR1.26, *p* = 0.085; TBS ≥ 9. HR 1.67, *p* = 0.005), and CA19‐9 level (> 40 U/mL. HR 1.49, *p* = 0.002) among the preoperative factors. Furthermore, preoperative chemotherapy and R1 resection were identified as significant perioperative factors (Table 2).

**TABLE 2:** Results of multivariate analysis of perioperative risk associated with overall survival after hepatectomy (*n* = 584).

Risk factors	Preoperative factors at diagnosis			Pre‐ and postoperative factors		
	HR	95% CI	*p*	HR	95% CI	*p*
Primary tumor nodal metastases						
No	1.00 (reference)			1.00 (reference)		
Yes	1.46	1.13–1.87	0.003	1.49	1.16–1.93	0.002
*K‐Ras* mutation status						
Wild type	1.00 (reference)			1.00 (reference)		
Mutated	1.51	1.21–1.89	< 0.001	1.53	1.22–1.92	< 0.001
Extra‐hepatic disease						
No	1.00 (reference)			1.00 (reference)		
Yes	1.8	1.37–2.36	< 0.001	1.70	1.29–2.25	< 0.001
Tumor burden score						
Low (< 3)	1.00 (reference)			1.00 (reference)		
Intermediate (3–8)	1.26	0.97–1.65	0.085	1.09	0.83–1.44	0.190
High (≥ 9)	1.67	1.17–2.39	0.005	1.3	0.87–1.94	0.190
CEA level (at diagnosis)						
≤ 20 ng/ml	1.00 (reference)			1.00 (reference)		
> 20 ng/ml	1.15	0.88–1.49	0.296	1.14	0.87–1.48	0.342
CA19‐9 level (at diagnosis)						
≤ 40 U/ml	1.00 (reference)			1.00 (reference)		
> 40 U/ml	1.49	1.15–1.93	0.002	1.47	1.13–1.91	0.004
Preoperative chemotherapy (before hepatectomy)						
No				1.00 (reference)		
Yes				1.29	1.02–1.63	0.028
Extent of liver resection						
Hr1 or less				1.00 (reference)		
Hr2 or larger				1.21	0.91–1.62	0.195
Curability and pathological resection margin						
R0 resection				1.00 (reference)		
Combined use of RFA				1.56	0.98–2.49	0.061
R1 resection				1.81	1.18–2.78	0.006
Blood transfusion						
No				1.00 (reference)		
Yes				1.19	0.88–1.61	0.240

Abbreviations: CA19‐9, carbohydrate antigen 19–9; CEA, carcinoembryonic antigen; CI, confidence interval; HR, hazard ratio; Hr1, sectionectomy; Hr2, hemihepatectomy; RFA, radiofrequency ablation.

**FIGURE 2:**
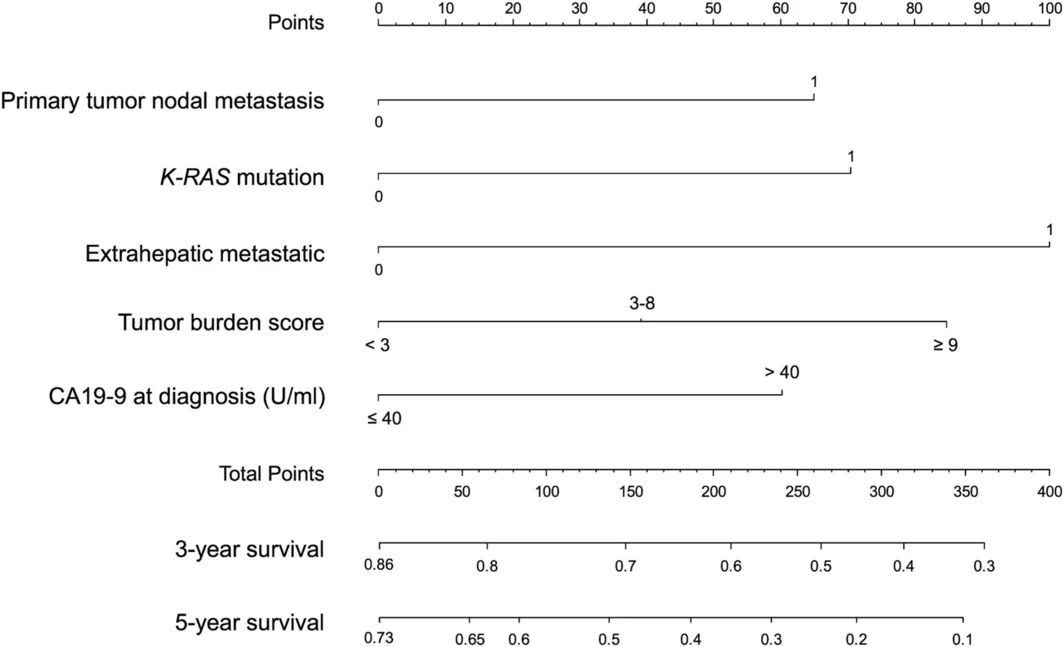
Nomogram for predicting 3‐ and 5‐year survival rates.

The above five independent prognostic factors recorded at the time of CRLM diagnosis were integrated to construct a prognostic nomogram for OS. The corresponding score for each variable can be obtained by drawing an upward vertical line on the score scale. To obtain the scores for each variable and sum them, to obtain the estimated rates for the 3‐ and 5‐year OS, this sum is placed on the “total points” axis and then a straight line is drawn perpendicular to the survival axis (Figure 2).

**FIGURE 3:**
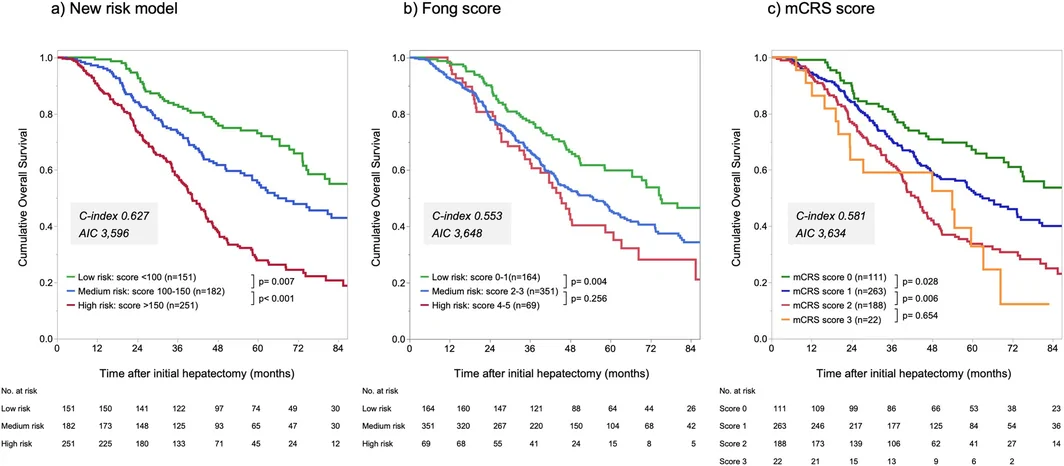
Kaplan–Meier survival curves for overall survival estimated by (a) new risk model, (b) Fong score, and (c) mCRS score.

Our cohort was classified into low‐risk (*n* = 151, total point [TP] < 100), medium‐risk (*n* = 182, TP 100–150), and high‐risk (*n* = 251, TP > 150) categories, resulting in a well‐stratified OS. The MST and 1−/3−/5‐OS were 95.6 months and 99.3/83.2/73.1% for the low‐risk, 66.0 months and 97.2/73.7/55.5% for the medium‐risk, 40.4 months and 90.4/57.8/27.9% for the high‐risk patients, respectively (Figure 3a). For comparison with the Fong score, the patients were divided into the low‐risk (Fong score 0–1), medium‐risk (Fong score 2–3) and high‐risk (Fong score 4–5) groups, as described previously [13]. Our risk model demonstrated significantly better discriminatory ability than the Fong score, with a Harrell's C‐index of 0.627 (95% confidence interval [CI], 0.598–0.656) versus 0.553 (95% CI, 0.524–0.581; *p* < 0.001) and a lower Akaike information criterion (AIC) (3596 vs. 3648) (Figure 3b). Likewise, our nomogram outperformed the modified clinical risk score (mCRS), which yielded a C‐index of 0.581 (95% CI, 0.550–0.613; *p* = 0.001) and an AIC of 3634 (Figure 3c). Internal validation of the Cox proportional hazards model demonstrated an apparent Harrell's C‐index of 0.644 (95% confidence interval [CI], 0.613–0.676) and an optimism‐corrected C‐index of 0.636 (bootstrap interval, 0.605–0.669). The apparent calibration slope was 1.000, and the optimism‐corrected calibration slope was 0.931, indicating minimal overfitting. Bootstrap calibration plots demonstrated good agreement between predicted and observed 3‐ and 5‐year overall survival probabilities (Figure S3).

**FIGURE 4:**
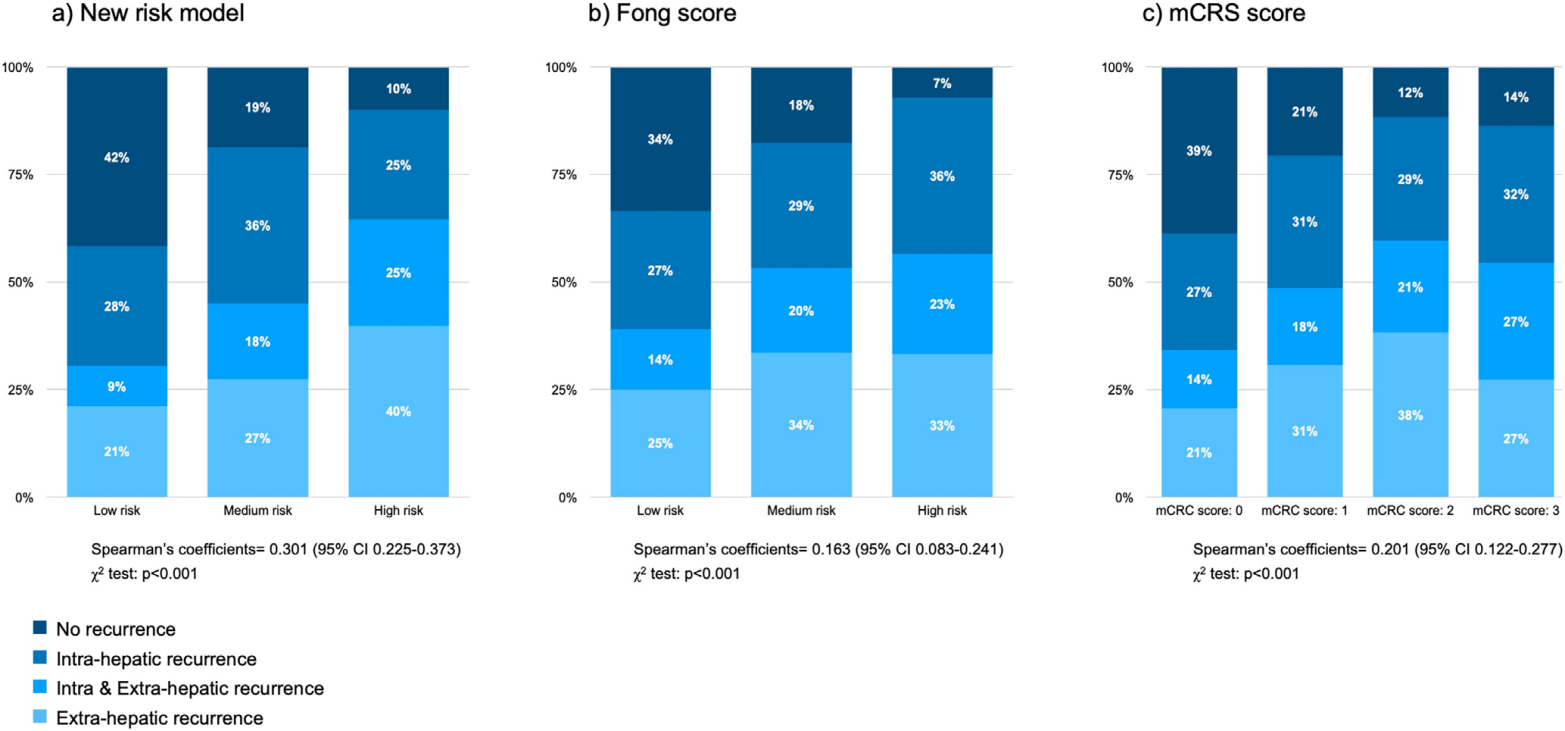
Recurrence pattern after initial hepatectomy in (a) our risk model, (b) Fong score, and (c) mCRS score.

Univariate and multivariate analyses of RFS identified the same preoperative risk factors as those identified in the OS analysis (Tables S2 and S3). When the nomogram‐derived risk groups were applied to RFS, they consistently stratified postoperative recurrence‐free survival. The MST and 1‐/3‐/5‐year RFS rates were 24.3 months and 72.2/46.4/42.1% in the low‐risk group, 11.4 months and 48.2/21.3/17.8% in the medium‐risk group, and 7.7 months and 31.1/9.9/7.9% in the high‐risk group, respectively (Figure S2a). The C‐index for our new model was 0.618 (95% CI; 0.592–0.643) versus 0.575 (95% CI 0.551–0.599, *p* = 0.002) for the Fong score. Consistent with these results, the AIC was lower for our risk model than the Fong score (5351 vs. 5398), indicating better discriminatory ability (Figure S2b). Moreover, it showed better discriminatory ability than the mCRS score, with a C‐index of 0.592 (95% CI; 0.565–0.617, *p* = 0.003) and AIC of 5389 (Figure S2c). Regarding recurrence patterns after initial hepatectomy, the proportion of extrahepatic recurrence, including lung, lymph node, and peritoneal metastasis, was increased by higher risk in each risk model (Figure 4). Our risk model well stratified the postoperative recurrence pattern compared with the traditional models; Spearman's coefficient was 0.301 (95% CI 0.225–0.373) in our model, 0.163 (95% CI 0.083–0.241) in the Fong score, and 0.201 (95% CI 0.122–0.277) in the mCRS score. Similarly, the discriminatory performances of our nomogram for OS and RFS were comparable to those of the GAME score and the JHBPS nomogram (Table S4).

## 4 Discussion

Recent advances in surgical and medical oncology have boosted efforts to identify markers associated with shorter OS and RFS in CRLM patients. In the current era of molecular targeted therapies, widespread molecular profiling of *the KRAS* gene has demonstrated its prognostic potential in CRLM [5, 14]. However, the prognostic impact of *KRAS* mutation can change depending on historical cohorts, as reported in one systematic review [15]. By analyzing data of patients who underwent genetic profiling, the present study evaluated the clinical features and prognostic impact of *KRAS* mutations in CRLM and established a new prognostic nomogram that included *KRAS* genetic profile.

Univariate and multivariate analyses showed that *KRAS* mutation has a negative impact on OS (HR 1.51, 95% CI 1.21–1.89) and RFS (HR 1.21, 95% CI 1.00–1.47). The prognostic values identified in our study are consistent with those described in the systematic review by Pikoulis et al. [15]. The authors of that review concluded that studies published before 2015 had higher cumulative HRs while later studies had significantly lower cumulative HRs. The same effect was found by stratifying the studies by sample size, with higher cumulative HRs observed in studies with fewer than 300 participants. This phenomenon could be attributed to either the development of diagnostic and treatment modalities or overestimation of clinical events in smaller studies. Our patient cohort comprised 584 patients who underwent initial liver resection between January 2006 and December 2016, had genetic profiles of *KRAS* mutations, and were followed‐up for a relatively long period. Thus, our study design and patient selection process ensured the validity and importance of the prognostic value of *KRAS* mutations in patients with CRLM.

The right and left sides of the colon are of embryologically different origin, with the right‐sided colon originating from the midgut while the left, including the rectum, originating from the hindgut. Therefore, adenocarcinomas of the left and right colon are associated with different molecular pathways of carcinogenesis [10, 16]. Our results showed the prevalence of *KRAS* mutations in patients with right‐sided primary CRC. Previous retrospective studies examined left–right differences in the primary tumor and the significance of this parameter as a prognostic factor for survival in patients with metastatic CRC, and the prevailing view is that right‐sided CRC is associated with poor survival [17]. For example, Arnold et al. [18] found that patients with right‐sided CRC had poor survival and response to anti‐EGFR treatment, irrespective of the chemotherapy regimen employed, and showed a poor response to anti‐EGFR treatment in patients with *RAS* wild‐type metastatic CRC. Nevertheless, Marques et al. [19] reported that sidedness did not affect long‐term survival of patients with resected colon cancer and liver metastases. Our survival analysis showed no significant difference in OS and RFS between right‐ and left‐sided primary lesions, both in the analysis of the entire group and analysis that focused on the impact of *KRAS* mutation (data not shown). We also examined the clinical implications of *KRAS* mutation profile stratified by tumor location. The results showed that the prognostic impact of *KRAS* mutation varied according to the colonic location of the primary tumor; CRLM with *KRAS* mutation were associated with poorer prognosis compared to those with wild‐type in left‐sided CRC. In contrast, among patients with right‐sided CRC, the prognosis was comparable between *KRAS* mutation and the wild type, suggesting no prognostic value for *KRAS* mutation. Our study also showed more frequent *KRAS* mutations in liver metastases from right‐sided primary tumors, confirming the previously reported data [6, 20]. Previous studies also demonstrated that *BRAF* mutations and microsatellite instability (MSI) are more frequent in right‐sided CRCs [21]. Further studies are needed to determine the biological characteristics that can define the prognosis of right‐sided colorectal cancer.

TBS is an established accurate tool to describe the impact of tumor morphology on long‐term survival in patients undergoing resection of CRLM [11]. European and Asian studies have also demonstrated the superiority of TBS compared to a variety of traditional morphological parameters, including tumor size and number. Another study considered the discriminatory power of imaging‐based TBS to be comparable to that of pathology‐based TBS [22]. In fact, univariate and multivariate analyses of our data did not show the superiority of tumor size and number as independent risk factors, compared with TBS. Therefore, TBS can be potentially used as an independent risk factor for other risks, including *KRAS* mutations. Interestingly, TBS grading discriminated postoperative OS in subgroup analysis of wild‐type patients. In contrast, OS for each TBS grade was comparable. In other words, *KRAS* mutation could define prognosis entirely independent of the morphological status of CRLM.

Prognostic models are essential tools for identifying patient subgroups with different prognoses. Moreover, they can be used in clinical decision‐making and prediction of treatment outcome. The Fong score was established in 1999 and is the most successfully used CRS in the surgical treatment of CRLM [9]. However, with recent advances in chemotherapy and surgical treatment for patients with CRLM, there is now concern that the Fong CRS does not adequately accommodate this in terms of its discriminatory power [23]. In this context, models that incorporate *KRAS* status have been developed to consider the biological grade of CRC, such as the Anderson modified CRS (mCRS) and the genetic and morphological evaluation (GAME) score [7, 8]. In this study, we created a new prognostic nomogram consisting of five clinical parameters. The five parameters identified in the multivariable analysis closely resembled those included in the GAME score, with CA19‐9 replacing CEA as the tumor marker component [8]. The predictive performance of the GAME score has been externally validated and represents an appropriate contemporary reference model for comparison with the present nomogram. Likewise, the JHBPS nomogram proposed by Beppu et al. represents an important prognostic model developed from a large Japanese multicenter cohort [24]. More recently, the RAS–Beppu classification has further integrated RAS mutation status into the Beppu classification for recurrence‐risk stratification [25].

In the present study, our nomogram demonstrated discriminatory performance comparable to that of both the GAME score and the JHBPS nomogram for predicting OS and RFS (Table S4). Importantly, however, the proposed model provides additional prognostic information beyond survival discrimination: the same biological risk framework consistently stratified both OS and RFS and was also associated with distinct postoperative recurrence patterns, including the risk of extrahepatic recurrence. These findings suggest that the proposed nomogram may capture broader aspects of tumor biology than survival prediction alone, while providing continuous biological risk assessment to support the concept of biological resectability. Although the RAS–Beppu classification was primarily developed for recurrence‐risk stratification, both approaches emphasize the importance of integrating tumor biology with conventional clinicopathological factors when assessing prognosis after hepatectomy for CRLM. First, in contrast to the use of CEA in the GAME, our nomogram selected CA19‐9 with a cut‐off value of 40 U/mL as a significant risk marker at the time of CRLM diagnosis. Recent CRLM‐specific studies have demonstrated the prognostic relevance of CA19‐9 in patients undergoing hepatectomy, including those treated with preoperative chemotherapy [26, 27]. In the present study, CA19‐9 was retained in the multivariable models for both OS and RFS, whereas CEA was not. Although the cutoff value of CA19‐9 was determined by ROC analysis within the present cohort, the resulting threshold (40 U/mL) was close to the commonly accepted upper limit of normal used in routine clinical practice. Therefore, the selected cutoff is considered clinically reasonable. Nevertheless, confirmation of the optimal cutoff value in independent cohorts will be important to establish its broader generalizability. Second, detailed point allocation was made in our nomogram for each risk factor to enhance prognostic prediction. Although the allocated GAME points are based on the hazard ratios estimated by the COX model, identical points are allocated for approximate hazard ratios: for example, *KRAS* mutation with HR 1.50 and CEA ≥ 20 with HR 1.90 are allocated the same “1 point”. Another example, extrahepatic disease with HR 2.10 and TBS ≥ 9 with HR 3.23 are assigned “2 points” and the same weighting [8]. Although the GAME score has the advantages of simplicity and accessibility, our nomogram preserves the relative weighting of individual prognostic factors and provides continuous risk estimation rather than categorical point assignment. Third, although the nomogram was originally developed for OS, the same preoperative variables independently predicted RFS, and the derived risk groups consistently stratified postoperative recurrence. Furthermore, the discriminatory performance of the model for RFS was comparable to that of the contemporary GAME score and JHBPS nomogram (Table S4). Thus, although originally developed for OS, the proposed biological risk framework demonstrated consistent prognostic relevance across both survival and recurrence outcomes. Beyond survival discrimination, the nomogram also stratified post‐hepatectomy recurrence patterns, with increasing biological risk associated with a greater proportion of extrahepatic recurrence. This finding is particularly relevant to the concept of biological resectability, because extrahepatic recurrence may reflect an underlying propensity for systemic metastatic spread rather than merely the anatomical burden of liver disease. In our cohort, extrahepatic recurrence was associated with poor post‐recurrence outcomes. Thus, the ability to stratify recurrence patterns may represent an additional clinically relevant feature of the model, potentially reflecting the propensity for systemic metastatic spread. The incorporation of molecular information may contribute to the ability of the model to stratify recurrence patterns. Recent genomic analyses have shown that co‐alterations involving oncogenic *Ras/Braf* and TP53 are associated with a greater propensity for extrahepatic metastatic spread [28]. These clinical observations are supported by preclinical evidence suggesting that interactions between TP53 loss and oncogenic *Ras/Braf* signaling promote invasion, motility, and metastatic progression [29, 30]. Taken together, these findings suggest that the proposed nomogram provides robust prognostic stratification across OS, RFS, and recurrence patterns while incorporating both molecular and clinicopathological determinants of metastatic behavior.

**FIGURE 5:**
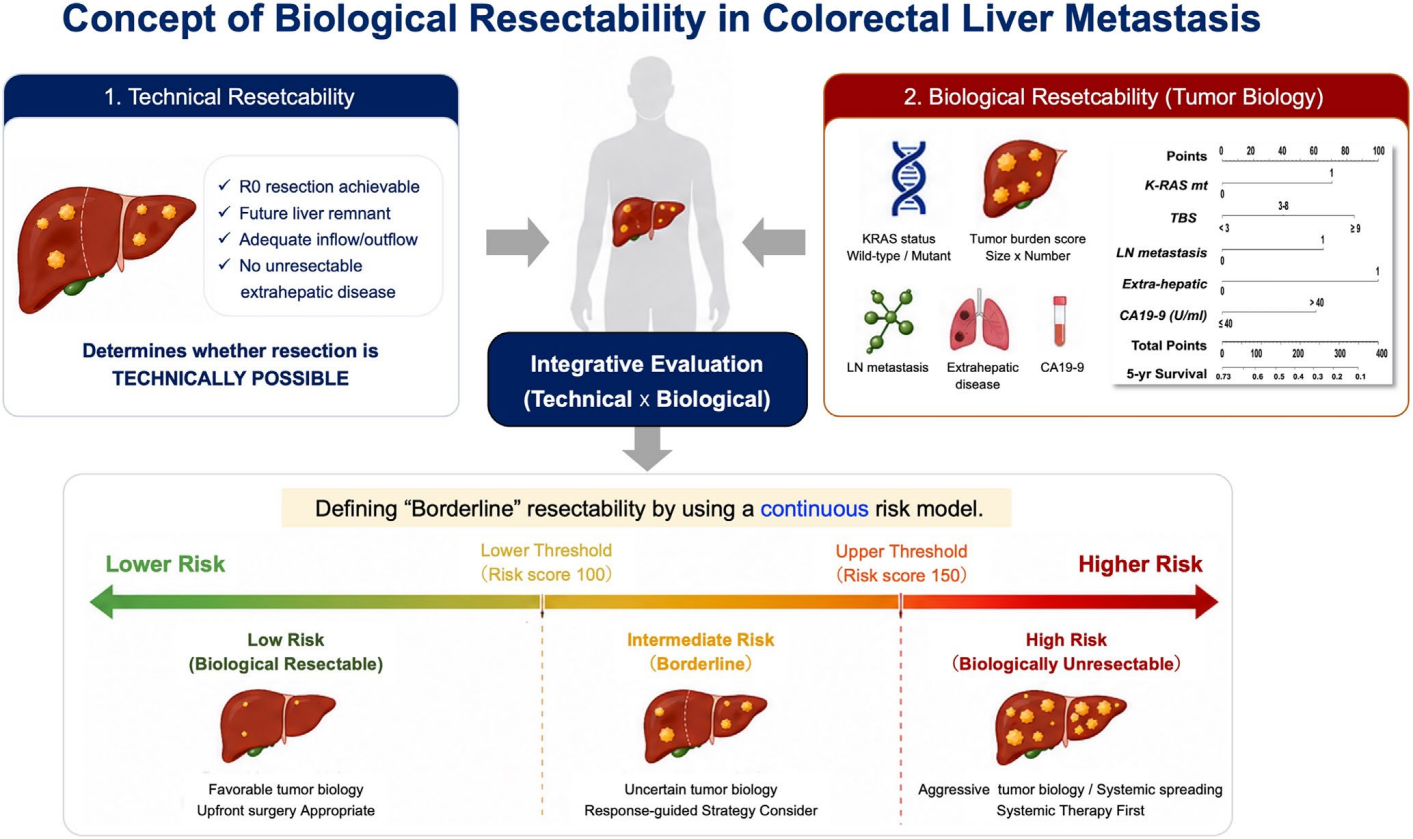
This schematic illustrates a hypothesis‐generating framework integrating technical resectability and tumor biology using the proposed nomogram. Continuous biological risk stratification may facilitate future investigations into biologically borderline resectable colorectal liver metastases. This framework is intended for conceptual illustration and has not been clinically validated as a treatment algorithm.

Interestingly, tumor recurrence was comparable in the low‐ and medium‐risk groups, indicating no difference in the control of recurrence with repeat hepatectomy or chemotherapy in these two groups. This may mean that these groups are distinct from the high‐risk group in terms of the degree of systemic cancer spread. In the high‐risk group, 90% of patients experienced recurrence after hepatic resection, meaning high probability of poor prognostic outcome due to poor response to treatment. In these cases, hepatic resection may only be a valid option in multimodal treatment and recurrence should not be labeled as an optimally resectable disease. From a prognostic perspective, it would be appropriate to distinguish patients with concomitant hepatic and extrahepatic lesions from those who meet the traditional resectable criteria to be considered “borderline resectable (BR)” [31]. This means that even if the tumor is technically resectable based on radiological imaging, one should carefully consider the risk of potential systemic cancer spread in BR‐CRLM with a high score on our nomogram. Importantly, because the present cohort included both technically resectable patients and patients who underwent conversion therapy following preoperative chemotherapy, the proposed concept of biological resectability should not be interpreted as a redefinition of technical resectability. Rather, our nomogram provides a continuous assessment of biological risk at the time of CRLM diagnosis and is intended to complement conventional anatomical assessment. Therefore, the proposed framework of biologically borderline resectable CRLM requires prospective validation, particularly in cohorts consisting predominantly of technically resectable patients. The conceptual framework illustrated in Figure 5 further emphasizes that resectability should be defined along a continuous biological spectrum rather than a binary classification. Our nomogram may therefore provide an objective approach to identify biologically borderline resectable CRLM and support more nuanced treatment decision‐making. Importantly, the proposed framework should be regarded as hypothesis‐generating and intended to facilitate future investigations into biological resectability rather than as a validated treatment algorithm for clinical decision‐making.

While we discussed above the advantages of our nomogram, one should consider the following limitations of our study. First, we generated the nomogram from a multicenter cohort of patients with CRLM. Because the nomogram was developed using variables derived from our multicenter cohort, its generalizability and comparative performance relative to existing prognostic models require confirmation in independent external cohorts. Although internal validation using bootstrap resampling demonstrated good calibration and only minimal optimism, the present nomogram was developed and evaluated within the same multicenter cohort. In addition, because *KRAS* testing was not routinely performed during the early study period, a substantial proportion of patients with unknown *KRAS* status were excluded from the analysis. This may have introduced selection bias toward a more contemporary molecularly characterized cohort with potentially different treatment strategies and clinical backgrounds. Moreover, because the proposed nomogram was designed to provide continuous estimation of biological risk, the cutoff values used for low‐, intermediate‐, and high‐risk stratification should be regarded as exploratory and primarily intended to facilitate clinical interpretation rather than to define fixed biological categories. Accordingly, further validation in independent contemporary cohorts, including international populations with different clinical practices, will be important to establish the generalizability of the present model beyond the Japanese multicenter cohort. Second, our risk model included *KRAS* mutations and lymph node metastasis from the primary tumor. It may be difficult to ascertain information on *KRAS* mutations and lymph node metastases preoperatively because the usefulness of the liver‐first approach has been advocated in recent years for synchronous liver metastases, particularly in advanced disease states [32]. However, it is possible to tentatively identify lymph node metastases with new radiological techniques, and *KRAS* mutations can be assessed in biopsies of the primary tumor. In addition, because the present nomogram was intentionally developed using variables available at the time of CRLM diagnosis, dynamic changes in tumor morphology, tumor marker levels, and radiological response following preoperative chemotherapy were not incorporated into the model. Although these variables may further refine biological risk assessment immediately before hepatectomy, they were not collected in a standardized manner across all participating institutions in this retrospective multicenter study. Future prospective studies should investigate whether incorporating treatment response during systemic therapy further improves the prediction of oncological outcomes. Lastly, although *KRAS* mutation analysis was performed using either primary colorectal tumor or liver metastasis specimens according to institutional practice, paired molecular analyses were not available. Therefore, the potential discordance of *KRAS* status between primary and metastatic lesions could not be evaluated, and its potential impact on prognostic stratification remains uncertain. In addition, in the early phase of the study, extended *RAS* analysis, including *NRAS* mutations, was not routinely performed because these alterations were not yet recognized as clinically relevant. Therefore, inadvertent inclusion of *NRAS* mutant tumors in the wild‐type cohort may have resulted in underestimation of the prognostic impact of *KRAS* mutations [33]. Furthermore, co‐alterations involving *TP53, SMAD4*, and other molecular factors were not evaluated and may also have influenced the observed outcomes [34].

In conclusion, we demonstrated that the prognostic impact of KRAS mutation depends on primary tumor sidedness and is independent of tumor morphology. Our nomogram demonstrated consistent prognostic relevance across OS and RFS and was associated with distinct postoperative recurrence patterns, including extrahepatic recurrence. These findings suggest that continuous biological risk assessment may facilitate individualized prognostic stratification and support future refinement of the concept of biological resectability in CRLM.

## Author Contributions

**Tatsuo Matsuda:** data curation. **Toru Kojima:** data curation. **Toshiyoshi Fujiwara:** data curation. **Tomokazu Fuji:** data curation. **Toshiharu Mitsuhashi:** methodology, data curation, software. **Takeshi Koujima:** data curation. **Masaru Inagaki:** data curation. **Yuzo Umeda:** conceptualization, methodology, data curation, formal analysis, writing – original draft, writing – review and editing, investigation, funding acquisition, visualization, project administration, resources, supervision, validation, software. **Daisuke Satoh:** data curation. **Kosei Takagi:** data curation.

## Funding

Financial support was received from the Okayama Medical Foundation and the Japan Society for the Promotion of Science (#22K08775 to Yuzo UMEDA).

## Ethics Statement

This study conformed to the Declaration of Helsinki on Human Research Ethics Standards and was approved by the Okayama University Hospital Institutional Ethics Board.

## Consent

The need for signed informed consent was waived due to the retrospective nature of the study.

## Conflicts of Interest

The authors declare no conflicts of interest.

## Supporting information


**Figure S1:** Kaplan–Meier survival curves stratified by *KRAS* mutation. (a) Overall survival, (b) Recurrence‐free survival.

**Figure S2:** Kaplan–Meier survival curves for recurrence‐free survival estimated by (a) new risk model, (b) Fong score, and (c) mCRS score.

**Figure S3:** Bootstrap calibration plots for prediction of overall survival. Calibration plots for 3‐year (A) and 5‐year (B) overall survival after hepatectomy for colorectal liver metastases. The dashed line represents the ideal calibration. The black solid line represents the apparent calibration in the original cohort, whereas the blue solid line represents the optimism‐corrected calibration based on 1000 bootstrap resamples. Gray solid lines indicate the 95% confidence intervals for the bias‐corrected calibration curve. Rug marks indicate the distribution of predicted survival probabilities.

**Table S1:** Background characteristics of the *K‐RAS* wild type and *K‐RAS* mutation groups.

**Table S2:** Results of univariate analysis of perioperative risk associated with recurrence‐free survival after hepatectomy (*n* = 584).

**Table S3:** Results of multivariate analysis of perioperative risk associated with recurrence‐free survival after hepatectomy (*n* = 584).

**Table S4:** Comparison of discriminatory performance among contemporary prognostic models.

## Data Availability

The data that support the findings of this study are available on request from the corresponding author. The data are not publicly available due to privacy or ethical restrictions.
