# Supplementary material for: Biological Resectability in Colorectal Liver Metastases: A Nomogram‐Based Continuous Risk Model to Define Borderline Disease
Source: Ann Gastroenterol Surg. 2026 Sep 8:10.1002/ags3.70278. Online ahead of print. doi: 10.1002/ags3.70278 (PMC13553971; doi:10.1002/ags3.70278)
Supplement: Supplementary file 1 [file AGS3-9999-0-s001.docx]

| **Supplementary Table 1.** Background characteristics of the *K-RAS* wild type and *K-RAS* mutation groups. | | | |
| --- | --- | --- | --- |
| Variables | *K-RAS* wild type (n=369) | *K-RAS* mutation (n=215) | P-value |
| Gender |  |  |  |
| female | 144 (39.0) | 99 (46.1) | 0.096 |
| male | 225 (61.0) | 116 (53.9) |  |
| Age |  |  |  |
| median (IQR) | 66 (59-71) | 67 (60-75) | 0.016 |
| ≤70 years | 265 (72.4) | 131 (61.5) | 0.007 |
| >70 years | 101 (27.6) | 82 (38.5) |  |
| Sidedness of primary colon cancer |  |  |  |
| right | 75 (20.3) | 72 (33.5) | <0.001 |
| left | 294 (79.7) | 143 (66.5) |  |
| Primary tumor nodal metastases |  |  |  |
| no | 129 (35.0) | 72 (33.5) | 0.718 |
| yes | 240 (65.0) | 143 (66.5) |  |
| Primary tumor differentiation |  |  |  |
| well differentiated | 69 (18.7) | 49 (22.8) | 0.446 |
| moderately differentiated | 268 (72.6) | 146 (67.9) |  |
| poorly differentiated/mucinous | 32 (8.7) | 20 (9.3) |  |
| Disease-free interval (months) |  |  |  |
| ≥12 | 122 (33.1) | 66 (30.7) | 0.555 |
| <12 | 247 (66.9) | 149 (69.3) |  |
| Extra-hepatic disease |  |  |  |
| no | 301 (81.6) | 177 (82.3) | 0.819 |
| yes | 68 (18.4) | 38 (17.7) |  |
| Tumor distibution |  |  |  |
| unilobar | 229 (62.1) | 143 (66.5) | 0.281 |
| bilobar | 140 (37.9) | 72 (33.5) |  |
| Number of tumors (at diagnosis of CRLM) | |  |  |
| median (IQR) | 2 (1-5) | 2 (1-4) | 0.176 |
| 1 | 144 (39.0) | 92 (42.8) | 0.313 |
| 2-4 | 131 (35.5) | 80 (37.2) |  |
| ≥5 | 94 (25.5) | 43 (20.0) |  |
| Maximum tumor diameter (at diagnosis of CRLM) | |  |  |
| median (IQR) | 2.7cm (1.7-4.5) | 2.5cm (1.7-4.0) | 0.314 |
| ≤5cm | 297 (80.5) | 181 (84.2) | 0.263 |
| >5cm | 72 (19.5) | 34 (15.8) |  |
| Tumor burden score |  |  |  |
| low (<3) | 101 (27.4) | 68 (31.6) | 0.214 |
| intermediate (3-8) | 207 (56.1) | 122 (56.7) |  |
| high (≥9) | 61 (16.5) | 25 (11.6) |  |
| CEA level (at diagnosis of CRLM) |  |  |  |
| ≤20 ng/ml | 237 (64.2) | 124 (57.7) | 0.116 |
| >20 ng/ml | 132 (35.8) | 91 (42.3) |  |
| CA19-9 level (at diagnosis of CRLM) | |  |  |
| ≤40 U/ml | 244 (66.1) | 143 (66.5) | 0.924 |
| >40 U/ml | 125 (33.9) | 72 (33.5) |  |
| Preoperative chemotherapy (before hepatectomy) | |  |  |
| no | 205 (55.6) | 135 (62.8) | 0.087 |
| yes | 164 (44.4) | 80 (37.2) |  |
| Basic regimen |  |  |  |
| singlet-FU base | 7 (4.3) | 7 (8.8) | 0.521 |
| doublet-OX base | 133 (81.1) | 60 (75.0) |  |
| doublet-CPT base | 19 (11.6) | 10 (12.5) |  |
| others | 5 (3.1) | 3 (3.8) |  |
| Biological agent |  |  |  |
| anti-EGFR | 56 (34.2) | 0 | <0.001 |
| anti-VEGF | 54 (32.9) | 53 (66.2) |  |
| none | 54 (32.9) | 27 (33.8) |  |
| Extent of liver resection |  |  |  |
| Hr1 or less | 289 (78.3) | 168 (78.1) | 0.959 |
| Hr2 or larger | 80 (21.7) | 47 (21.9) |  |
| Open/Laparoscopic procedure |  |  |  |
| open | 329 (89.2) | 186 (86.5) | 0.339 |
| laparoscopic | 40 (10.8) | 29 (13.5) |  |
| Curability and pathological resection margin | |  |  |
| R0 resection | 330 (89.4) | 190 (88.4) | 0.484 |
| combined use of RFA | 18 (4.9) | 8 (3.7) |  |
| R1 resection | 21 (5.7) | 17 (7.9) |  |
| Blood loss |  |  |  |
| median (IQR) | 405 (200-703) | 325 (150-640) | 0.015 |
| ≤1000 ml | 306 (82.9) | 191 (88.8) | 0.049 |
| >1000 ml | 63 (17.1) | 24 (11.2) |  |
| Blood transfusion |  |  |  |
| no | 304 (82.4) | 184 (85.6) | 0.315 |
| yes | 65 (17.6) | 31 (14.4) |  |
| Adjuvant chemotherapy (after hepatectomy) | |  |  |
| no | 150 (40.6) | 102 (47.4) | 0.110 |
| yes | 219 (59.4) | 113 (52.6) |  |

**Abbreviations.** CEA. carcinoembryonic antigen; CA19-9. carbohydrate antigen 19-9; CPT. irinotecan; CRLM. colorectal liver metastasis; EGFR. epidermal growth factor receptor; FU. fluorouracil; IQR. interquartile range; Hr1. sectionectomy; Hr2. hemihepatectomy; OX. oxaliplatin; RFA. radiofrequency ablation; VEGF. vascular endothelial growth factor
