# Supplementary material for: Biological Resectability in Colorectal Liver Metastases: A Nomogram‐Based Continuous Risk Model to Define Borderline Disease
Source: Ann Gastroenterol Surg. 2026 Sep 8:10.1002/ags3.70278. Online ahead of print. doi: 10.1002/ags3.70278 (PMC13553971; doi:10.1002/ags3.70278)
Supplement: Supplementary file 2 [file AGS3-9999-0-s003.docx]

| **Supplementary Table 4.** Comparison of discriminatory performance among contemporary prognostic models. | | | | | | | | | | |
| --- | --- | --- | --- | --- | --- | --- | --- | --- | --- | --- |
| Risk model | Overall survival | | | | | Recurrence-free survival | | | | |
|  | AIC | BIC | Harrell’s C-index | 95% CI | P-value* | AIC | BIC | Harrell’s C-index | 95% CI | P-value* |
| New risk model | 3595.5 | 3604.2 | 0.627 | 0.598-0.656 | - | 5351.4 | 5360.1 | 0.618 | 0.592-0.643 | - |
| GAME score | 3606.8 | 3615.5 | 0.613 | 0.584-0.642 | 0.186 | 5359.4 | 5368.1 | 0.614 | 0.590-0.639 | 0.702 |
| JHBPS nomogram | 3620.8 | 3638.3 | 0.605 | 0.574-0.636 | 0.162 | 5372.7 | 5390.2 | 0.609 | 0.583-0.634 | 0.489 |
| mCRS score | 3634.4 | 3647.4 | 0.581 | 0.550-0.613 | 0.001 | 5389.4 | 5407.1 | 0.592 | 0.565-0.617 | 0.003 |
| Fong score | 3648.0 | 3656.7 | 0.553 | 0.524-0.581 | <0.001 | 5398.3 | 5407.1 | 0.575 | 0.551-0.599 | 0.019 |
| **Abbreviations.** AIC. Akaike's Information Criterion; BIC. Bayesian Information Criterion; CI. confidence interval; GAME. Genetic and Morphological Evaluation; mCRS. MD Anderson modified clinical risk score  * P values compare Harrell’s C-index of the proposed nomogram with that of each reference model, accounting for within-cohort covariance. | | | | | | | | | | |
