# Supplementary material for: Biological Resectability in Colorectal Liver Metastases: A Nomogram‐Based Continuous Risk Model to Define Borderline Disease
Source: Ann Gastroenterol Surg. 2026 Sep 8:10.1002/ags3.70278. Online ahead of print. doi: 10.1002/ags3.70278 (PMC13553971; doi:10.1002/ags3.70278)
Supplement: Supplementary file 3 [file AGS3-9999-0-s006.docx]

| **Supplementary Table 3.** Results of multivariate analysis of perioperative risk associated with recurrence-free survival after hepatectomy (n=584). | | | | | | | |
| --- | --- | --- | --- | --- | --- | --- | --- |
| Risk factors | Preoperative factors at diagnosis | | | | Pre- and postoperative factors | | |
|  | HR | | 95% CI | P-value | HR | 95% CI | P-value |
| Primary tumor nodal metastases |  | |  |  |  |  |  |
| no | 1.00 (reference) | |  |  | 1.00 (reference) |  |  |
| yes | 1.55 | | 1.25-1.91 | <0.001 | 1.63 | 1.33-1.99 | <0.001 |
| Primary tumor differentiation |  | |  |  |  |  |  |
| well differentiated | 1.00 (reference) | |  |  | 1.00 (reference) |  |  |
| moderately differentiated | 1.21 | | 0.97-1.52 | 0.113 | 1.29 | 1.02-1.64 | 0.034 |
| poorly differentiated/mucinous | 1.27 | | 0.89-1.84 | 0.187 | 1.44 | 0.99-2.09 | 0.051 |
| *K-Ras* mutation status |  | |  |  |  |  |  |
| wild type | 1.00 (reference) | |  |  | 1.00 (reference) |  |  |
| mutated | 1.21 | | 1.00-1.46 | 0.045 | 1.16 | 0.97-1.41 | 0.100 |
| Disease-free interval (months) |  | |  |  |  |  |  |
| ≥12 | 1.00 (reference) | |  |  | 1.00 (reference) |  |  |
| <12 | 1.13 | | 0.92-1.40 | 0.227 | 1.19 | 0.90-1.38 | 0.306 |
| Extra-hepatic disease |  | |  |  |  |  |  |
| no | 1.00 (reference) | |  |  | 1.00 (reference) |  |  |
| yes | 1.51 | | 1.20-1.91 | <0.001 | 1.51 | 1.19-1.90 | <0.001 |
| Tumor burden score |  | |  |  |  |  |  |
| low (<3) | 1.00 (reference) | |  |  | 1.00 (reference) |  |  |
| intermediate (3-8) | 1.39 | | 1.11-1.77 | 0.005 | 1.34 | 1.05-1.70 | 0.016 |
| high (≥9) | 2.16 | | 1.54-3.03 | <0.001 | 2.13 | 1.47-3.09 | <0.001 |
| CEA level (at diagnosis of CRLM) |  | |  |  |  |  |  |
| ≤20 ng/ml | 1.00 (reference) | |  |  | 1.00 (reference) |  |  |
| >20 ng/ml | 1.02 | | 0.83-1.27 | 0.776 | 1.03 | 0.83-1.28 | 0.740 |
| CA19-9 level (at diagnosis of CRLM) |  | |  |  |  |  |  |
| ≤40 U/ml | 1.00 (reference) | |  |  | 1.00 (reference) |  |  |
| >40 U/ml | 1.34 | | 1.08-1.66 | 0.007 | 1.21 | 0.97-1.50 | 0.065 |
| Preoperative chemotherapy (before hepatectomy) |  | |  |  |  |  |  |
| no |  | |  |  | 1.00 (reference) |  |  |
| yes |  | |  |  | 1.2 | 0.98-1.46 | 0.065 |
| Extent of liver resection |  | |  |  |  |  |  |
| Hr1 or less |  | |  |  | 1.00 (reference) |  |  |
| Hr2 or larger |  | |  |  | 1.07 | 0.84-1.37 | 0.560 |
| Curability and pathological resection margin |  | |  |  |  |  |  |
| R0 resection |  | |  |  | 1.00 (reference) |  |  |
| combined use of RFA |  | |  |  | 1.40 | 0.90-2.18 | 0.132 |
| R1 resection |  | |  |  | 1.92 | 1.35-2.74 | <0.001 |
| Adjuvant chemotherapy (after hepatectomy) |  | |  |  |  |  |  |
| no |  |  | |  | 1.00 (reference) |  |  |
| yes |  |  | |  | 0.63 | 0.52-0.76 | <0.001 |
| **Abbreviations.** CEA. carcinoembryonic antigen; CA19-9. carbohydrate antigen 19-9; CI. confidence interval; HR. hazard ratio; Hr1. sectionectomy; Hr2. hemihepatectomy; RFA. Radiofrequency ablation; | | | | | | | |
