# Supplementary material for: Biological Resectability in Colorectal Liver Metastases: A Nomogram‐Based Continuous Risk Model to Define Borderline Disease
Source: Ann Gastroenterol Surg. 2026 Sep 8:10.1002/ags3.70278. Online ahead of print. doi: 10.1002/ags3.70278 (PMC13553971; doi:10.1002/ags3.70278)
Supplement: Supplementary file 4 [file AGS3-9999-0-s007.docx]

**Supplementary Table 2.** Results of univariate analysis of perioperative risk associated with recurrence-free survival after hepatectomy (n=584).

| Risk factors | n (%) | Recurrence-free survival | | Univariate analysis | | |
| --- | --- | --- | --- | --- | --- | --- |
|  |  | 5 years (%) | Median (month) | HR | 95% CI | P-value |
| Gender |  |  |  |  |  |  |
| female | 243 (41.6) | 19.9 | 10.5 | 1.00 (reference) |  |  |
| male | 341 (58.4) | 19.7 | 11.6 | 0.85 | 0.68-1.07 | 0.180 |
| Age |  |  |  |  |  |  |
| ≤70 years | 398 (68.2) | 22.0 | 11.4 | 1.00 (reference) |  |  |
| >70 years | 186 (31.8) | 15.2 | 10.3 | 1.14 | 0.94-1.38 | 0.176 |
| Sidedness of primary colon cancer |  |  |  |  |  |  |
| right | 147 (25.2) | 22.5 | 12.0 | 1.00 (reference) |  |  |
| left | 437 (74.8) | 19.1 | 10.8 | 1.03 | 0.84-1.27 | 0.746 |
| Primary tumor nodal metastases |  |  |  |  |  |  |
| no | 201 (34.4) | 31.8 | 14.8 | 1.00 (reference) |  |  |
| yes | 383 (65.6) | 13.6 | 9.2 | 1.63 | 1.28-2.09 | <0.001 |
| Primary tumor differentiation |  |  |  |  |  |  |
| well differentiated | 118 (20.2) | 26.3 | 13.8 | 1.00 (reference) |  |  |
| moderately differentiated | 414 (70.9) | 18.4 | 10.6 | 1.24 | 0.99-1.58 | 0.061 |
| poorly differentiated/mucinous | 52 (8.9) | 17.9 | 7.6 | 1.41 | 0.98-1.58 | 0.062 |
| *K-Ras* mutation status |  |  |  |  |  |  |
| wild type | 369 (63.2) | 21.9 | 12.4 | 1.00 (reference) |  |  |
| mutated | 215 (36.8) | 16.3 | 9.6 | 1.21 | 1.00-1.47 | 0.044 |
| Disease-free interval (months) |  |  |  |  |  |  |
| ≥12 | 188 (32.2) | 25.9 | 13.5 | 1.00 (reference) |  |  |
| <12 | 396 (67.8) | 17.1 | 9.6 | 1.37 | 1.13-1.67 | 0.002 |
| Extra-hepatic disease |  |  |  |  |  |  |
| no | 478 (81.8) | 22.7 | 12.0 | 1.00 (reference) |  |  |
| yes | 106 (18.2) | 6.9 | 7.8 | 2.06 | 1.59-2.69 | <0.001 |
| Uni- or bilobar disease |  |  |  |  |  |  |
| unilobar | 372 (63.7) | 22.2 | 12.0 | 1.00 (reference) |  |  |
| bilobar | 212 (36.3) | 15.9 | 8.9 | 1.28 | 1.06-1.54 | 0.009 |
| Tumor burden score |  |  |  |  |  |  |
| low (<3) | 169 (28.9) | 32.6 | 17.6 | 1.00 (reference) |  |  |
| intermediate (3-8) | 329 (56.3) | 17.5 | 10.6 | 1.51 | 1.22-1.88 | <0.001 |
| high (≥9) | 86 (14.7) | 4.2 | 6.0 | 2.74 | 2.05-3.64 | <0.001 |
| CEA level (at diagnosis of CRLM) |  |  |  |  |  |  |
| ≤20 ng/ml | 361 (61.8) | 23.1 | 12.2 | 1.00 (reference) |  |  |
| >20 ng/ml | 223 (38.2) | 14.7 | 8.6 | 1.37 | 1.14-1.64 | <0.001 |
| CA19-9 level (at diagnosis of CRLM) |  |  |  |  |  |  |
| ≤40 U/ml | 387 (66.3) | 24.4 | 12.2 | 1.00 (reference) |  |  |
| >40 U/ml | 197 (33.7) | 11.2 | 7.9 | 1.55 | 1.29-1.87 | <0.001 |
| Preoperative chemotherapy (before hepatectomy) | |  |  |  |  |  |
| no | 340 (58.2) | 23.2 | 12.4 | 1.00 (reference) |  |  |
| yes | 244 (41.8) | 15.3 | 8.6 | 1.34 | 1.12-1.62 | 0.001 |
| Extent of liver resection |  |  |  |  |  |  |
| Hr1 or less | 457 (78.3) | 22.1 | 12.0 | 1.00 (reference) |  |  |
| Hr2 or larger | 127 (21.7) | 12.0 | 7.8 | 1.45 | 1.17-1.80 | <0.001 |
| Open/Laparoscopic procedure |  |  |  |  |  |  |
| open | 515 (88.2) | 19.2 | 10.5 | 1.00 (reference) |  |  |
| laparoscopic | 69 (11.8) | 25.1 | 13.3 | 0.78 | 0.59-1.05 | 0.111 |
| Curability and pathological resection margin |  |  |  |  |  |  |
| R0 resection | 520 (89.0) | 21.6 | 11.5 | 1.00 (reference) |  |  |
| combined use of RFA | 26 (4.5) | 11.5 | 6.9 | 1.49 | 0.99-2.26 | 0.054 |
| R1 resection | 38 (6.5) | 2.6 | 6.1 | 2.01 | 1.43-2.82 | <0.001 |
| Blood loss |  |  |  |  |  |  |
| ≤1000 ml | 497 (85.1) | 20.1 | 11.1 | 1.00 (reference) |  |  |
| >1000 ml | 87 (14.9) | 18.7 | 9.2 | 1.18 | 0.87-1.59 | 0.274 |
| Blood transfusion |  |  |  |  |  |  |
| no | 488 (83.6) | 20.0 | 10.9 | 1.00 (reference) |  |  |
| yes | 96 (16.4) | 19.4 | 9.9 | 1.03 | 0.81-1.32 | 0.770 |
| Adjuvant chemotherapy (after hepatectomy) |  |  |  |  |  |  |
| no | 252 (43.2) | 15.5 | 8.4 | 1.00 (reference) |  |  |
| yes | 332 (56.8) | 23.3 | 12.8 | 0.69 | 0.58-0.84 | <0.001 |

**Abbreviations.** CEA. carcinoembryonic antigen; CA19-9. carbohydrate antigen 19-9; CI. confidence interval; HR. hazard ratio; Hr1. sectionectomy; Hr2. hemihepatectomy; RFA. radiofrequency ablation
